# Personalizing a mental health texting intervention using reinforcement learning

**DOI:** 10.1038/s44184-025-00173-3

**Published:** 2025-12-02

**Authors:** Marvyn R. Arévalo Avalos, Karina Rosales, Chris Karr, Caroline A. Figueroa, Tiffany Luo, Suchitra Sudarshan, Vivian Yip, Adrian Aguilera

**Affiliations:** 1 School of Social Welfare, University of California Berkeley, Berkeley, CA USA; 2 Audacious Software, Chicago, IL USA; 3 Faculty of Technology, Policy, and Management, Delft Technical University, Delft, The Netherlands; 4 Department of Psychiatry and Behavioral Sciences, University of California–San Francisco, San Francisco, CA USA

**Keywords:** Anxiety, Depression

## Abstract

StayWell is a 60-day CBT/DBT-based text messaging intervention which leverages reinforcement learning algorithms to support mental health. Participants were randomly assigned to receiving personalized messaging (adaptive arm), static messaging (random arm) or mood-monitoring only messages (control arm). A diverse sample of 1121 adults participated in a fully remote trial between December 2021 and July 2022. Across study arms, participants showed a 25% reduction in depression symptoms (PHQ-8) and 24% reduction in anxiety symptoms (GAD-7) following the intervention. We did not find statistically significant differences in PHQ-8 and GAD-7 reductions between intervention arms. Participants in the control arm had higher mood-monitoring messages response rates than those in other conditions. Finally, post-hoc exploratory analysis assessing outcomes by condition indicated that patients with minimal to mild depression symptoms (PHQ-8 < 10) benefitted from the reinforcement learning algorithm. The results of this trial suggest that StayWell is a promising text-messaging intervention to achieve reductions in depression and anxiety among diverse populations.

## Introduction

Depression and anxiety are pervasive mental health conditions that have negatively impacted diverse populations in the United States, with symptoms rising dramatically since the advent of the COVID-19 pandemic^1^. Despite the prevalence of these conditions, both treatment and preventative programs do not meet the large existing need for intervention. Current evidence-based treatments based in Cognitive Behavioral Therapy (CBT) and Dialectical Behavior Therapy (DBT) have proven successful in reducing depression and anxiety symptoms^2,3^, but a significant portion of those affected remain untreated. Access to care can be attributed to factors, such as stigma, cost, time constraints, provider availability and difficulties navigating complex mental health systems in the U.S^4^.

Emerging research indicates that digital interventions may help bridge the gap by offering accessible, evidence-based tools to manage or prevent symptoms of depression and anxiety on a public health scale. Particularly, text messaging programs have been shown to effectively reduce symptoms of depression and anxiety^5^ and can reach communities that are difficult to access, such as racial and ethnic minoritized groups^6^. Moreover, these programs have been employed during times of crisis, such as the COVID-19 pandemic^7,8^, offering support to wide-reaching audiences.

However, one prominent limitation of such programs is their static design, which does not personalize to users’ individual needs and preferences. This lack of personalization leads to low engagement, associated with low effectiveness. The just in time adaptive intervention (JITAI) approach^9,10^ seeks to improve upon static interventions by sending the optimal intervention at the right time. Machine learning techniques, such as reinforcement learning algorithms can power a JITAI approach by personalizing text message content and timing based on the user’s response to the intervention^10^. This approach has been applied to health interventions including increasing physical activity^11^ and improving medication adherence^12^ with increased personalization linked with greater efficacy and user satisfaction^13,14^. A reinforcement learning algorithm, in the context of a mental health text messaging intervention, is an AI-driven system that learns over time to personalize message delivery by optimizing the type, timing, and sequence of intervention messages based on a key outcome variable, such as daily mood rating, with the goal of maximizing high mood ratings. This could potentially lead to more effective interventions that are responsive to the needs and preferences of users.

The StayWell Program is a 60-day text-messaging intervention designed to provide mental health support tips and mood monitoring via daily text messages to users^7^. In the first iteration of the StayWell at Home texting program deployed during the beginning of the COVID pandemic, participants observed significant reductions in PHQ-9 and GAD-2 symptoms after 60 days of use^7^. In the second iteration of the Staywell program, we found that the Latinx population experienced a more significant decrease in depression and anxiety symptoms over the course of the program than non-Latinx white participants^8^. Building on these findings, the present study aims to further increase personalization by testing a reinforcement learning algorithm that could potentially optimize mood improvement via better message selection. By applying reinforcement learning to make decisions on content delivery in a text messaging intervention, we hope to provide a more personalized and effective intervention strategy for those experience symptoms of depression and anxiety.

In this article, we outline the results of a trial examining the efficacy of StayWell with reinforcement learning, a CBT/DBT-based text messaging program to support mental health. The StayWell program leverages reinforcement learning algorithms (Adaptive study arm—personalization) with the goal of reducing depression and anxiety symptoms compared to a random message group (Random study arm—static, no personalization) and a mood monitoring control group (Control arm). We hypothesized:

1. Participants in StayWell program, across all three arms of the study, would experience reductions in depression and anxiety symptoms.
2. Reductions in depression and anxiety symptoms would be greatest for participants in the Adaptive arm of the study, followed by those in the Random arm, and finally the control arm.
3. Engagement measured by mood-prompt response rates would be highest for the Adaptive group, followed by the Random, and finally the Control group.

Hypothesis 1 is grounded in previous findings from our research team in which earlier versions of the StayWell at Home program indicated improvements in mental health symptoms for participants. Hypothesis 2 and 3 are grounded in research indicating personalization is related to greater efficacy and user satisfaction.

## Methods

### StayWell trial design

The StayWell randomized controlled trial was fully remote and took place between December 2021 and June 2022. The intervention included three arms: an adaptive arm, a random arm, and a control text-messaging arm. The institutional review board of the University of California (UC) Berkeley reviewed and approved all study procedures and the trial was pre-registered at ClinicalTrials.gov (NCT04473599).

### Participants and recruitment

Participants were recruited primarily via web-based advertisements posted on social media sites (e.g., Facebook, Instagram) and via email invitations sent through the HealthMatch Clinical Trials patient portal (https://healthmatch.io/). English and Spanish language Facebook advertisements were created with the Facebook Ads Manager tool, featuring images of people using mobile devices, a caption that called for volunteers interested in mental health, and a link to a Qualtrics baseline questionnaire to enroll in the study. Ads were customized to target working class adults aged 22–55 years, who were living in the United States and had an interest in mental health. A daily budget of $15 was used for Facebook advertisements. The total budget spent on FB advertisement was $2382.96. We also partnered with HealthMatch to recruit via their patient portal, which added a sample with higher symptoms to increase our ability make comparisons based on symptom level. HealthMatch pre-screens potential research participants based on self-reported symptoms/diagnosis and matches them to existing registered Clinical Trials. Patients who expressed interest in mental health via the HealthMatch portal were sent individualized emails with the study baseline questionnaire inviting them to participate in the study. Study eligibility included residing in the U.S., 18 years of age or older, English or Spanish speakers, completing a baseline questionnaire, having a mobile phone, and registering using a U.S. based cell phone number. Participants were excluded if they provided incomplete baseline data or registered with an internet-based phone number (e.g., Google Voice) as these users were deemed to be associated with scammer/spamming accounts.

### Procedures

Participants who agreed to the informed consent documentation administered via Qualtrics before the baseline survey, completed the baseline survey, and passed phone verification procedures were automatically enrolled in the StayWell program via our secure text-messaging platform, HealthySMS. Randomization was conducted automatically within HealthySMS to ensure a balanced allocation into the three intervention arms (block randomization 1:1:1), stratified by participant language. Interested subjects consented to our study and answered questions pertaining to their demographics and other measures of interest through a Qualtrics survey. The survey contained a validation question (A multiple choice question: “What is the first letter of this question?” with the correct answer being “W”) and a built-in *captcha* in order to ensure that surveys were not completed by web-based scams and/or fraud. On day 61 of participants’ enrollment, they received an automated text message from HealthySMS with a link to complete an exit survey via Qualtrics. Participants had 14 days to complete the survey. If the survey was not complete at day 14, a reminder text message was manually sent from HealthySMS to participants. Upon completion of the exit survey, participants were paid US $20.

### Intervention

The StayWell Program is a 60-day text-messaging intervention designed to provide its users (adults ages 18 + ) with psychoeducation and mental health support tips as well as mood monitoring via delivery of daily text messages. The mental health tip text messages were adapted and expanded upon by the first author, MAA, based on an earlier version of the StayWell at Home program designed to counteract depression and anxiety during the COVID-19 social distance mandates^7,8^. A total of 250 messages were created to represent six distinct categories of CBT/DBT interventions (i.e., Accumulating Positives “-ap-”, Building Mastery “-bm-“, Distress Tolerance “-dt-“, Helpful Thinking “-ht-“, Interpersonal Effectiveness “-ie-“, and PLEASE Skills “-pl-“). The co-authors KR, SS, VY, and a research volunteer reviewed the messages for clarity and representation of the message into each of the six distinct CBT/DBT intervention categories. Messages that were unclear, ambiguous, or did not fit into a single category were removed. Finally, two bilingual and bicultural co-authors MAA and KR translated the messages into Spanish to expand the reach of the intervention. The final message bank included 192 messages, each representing the six distinct and mutually exclusive category of CBT/DBT interventions and a “no message” category. Message category definitions and sample messages are presented in Table 1. After receiving the mental health tip message, all participants received a daily mood-prompt text message (“What is your mood right now from 1 to 9?”) three hours after the delivery of the mental health tip message. A mood-reminder text message (“Please don’t forget to send in your mood rating”) was delivered 50% of the time to participants if they did not respond within two hours of the first mood-prompt message. After responding with a mood rating, a mood-feedback message providing general reflection based on mood level (low, mid, high mood) was delivered either 33, 66, or 100% of the time to assess differences in response rates based on feedback level as an exploratory aim. The messages sent to participants were scheduled to be sent out daily at random times between 9 AM and 6 PM.

**Table 1:** Message Categories and Descriptive Statistics by Intervention Arm

Message Category	Message Definition	Sample Text Message	Distinct messages	Random			Adaptive		
				Mode	Mean	Min, Max	Mode	Mean	Min, Max
Accumulating positives	Engaging in activities that can lead to pleasant or enjoyable experiences and emotions.	• Finding ways to play and laugh is super important for our mental and physical health • Missing someone you love? Write a letter or a little note to someone you haven’t talked to in a while.	33	7	8.10	2, 16	6	8.34	1, 18
Building mastery	Engaging in activities that increase a sense of self-efficacy, self-control, and competence.	• Keeping a routine is key. Set a time to work and time to take breaks • Procrastinating on a task? Break it up into smaller sections and reward yourself with your favorite snack or activity as you achieve each step!	32	8	8.45	2, 18	9	7.98	3, 16
Distress tolerance	These are a set of skills (e.g., self-soothing, radical acceptance, distracting the mind) used to tolerate and accept difficult or distressing situations. These skills require a person to observe one’s environment/emotional situation and to recognize what is happening without the intention to change/control the situation.	• It’s ok to feel anxious! Breathe in for 3 seconds…. out for 3 seconds. • Use mindfulness or other stress reduction tools if worry is keeping you up at night	33	10	8.48	3, 15	8	8.17	1, 15
Helpful thinking	These skills encourage others to actively engage in thinking positively about their situation, to remember helpful thoughts, and/or to reframe negative thinking with more balanced/positive thoughts	• Try to focus on the facts about a situation and not jump to conclusions too quickly. • What are three things that you are thankful for?	31	7	8.22	1, 18	8	8.26	2, 16
Interpersonal effectiveness	These skills promote behaviors/attitudes that help participants improve their existing relationships or manage/cope with unhelpful relationships.	• Recognize your personal boundaries and communicate assertively with others if you feel disrespected. • Remember that relationships are a two-way street, give some and take some.	33	9	8.27	2, 16	10	8.44	2, 16
PLEASE skills	PLEASE skills focus on taking care of one’s body in order to reduce emotional vulnerability. The PLEASE acronym stands for: **PL -** treat Physical Illness **E -** Eating balanced **A** - Avoid Mood Altering Drugs **S** - Balance Sleep **E** - Get Exercise	• Exercising can improve mood, reduce anxiety, and boost focus. Time to get those endorphins and positive energy going! • Excessive alcohol or drug use may negatively impact your energy and mood.	30	8	8.40	1, 17	9	8.21	2, 16
No message	No tip message sent	• n/a	1	8	8.35	2, 16	9	8.16	1, 15

^*^n/a not applicable.

### Adverse events

The research team programmed “keyword alerts” in English and Spanish within the HealthySMS system designed to assess patient’s potential endorsement of danger to self or danger to others. The alerts were logged in the HealthySMS system and sent via automatic emails to the research team. The alerts were triggered if the participant interacted (e.g., liked a message) or responded to a message containing any of the keywords, such as: “Kill, Die, Suicide, Bridge, Harm, Hurt, or Gun” and their corresponding Spanish-language words. The automatic emails and corresponding message logs were manually reviewed by research staff members within 24-hours of receiving an alert; and if necessary, the team coordinated a respond to the participant for additional assessment and resource sharing.

### Intervention arms

Participants in the Adaptive arm of the study received daily messages as chosen by the reinforcement learning algorithm. The StayWell messaging algorithm used individualized linear regression models to attempt to send messages that optimize the reported mood rating score by choosing the optimal parameters: A) Mental health support tip message selected from the previously described categories: “-ap-“, “-bm-“, “-dt-“, “-ht-“, “-ie-“, “-pl-“, and “-none-“, where a “none” message indicates that no message will go out that day. B) Time of day, selected from a pre-set list of time windows: 9 AM–12 PM, 12 PM–3 PM, 3 PM–6 PM. Every morning the reinforcement learning updated the regression models based on the following variables: day of the week, whether the day is a weekday or weekend, average mood rating over the past week, which message category was previously sent and at what time, and days since a message from that particular category type was sent.

On the other hand, the participants in the Random arm of the study received messages at random for each of the message category types and the timing of message. Thus, each day each participant had a 0.143 probability of receiving a message from the categories listed above and a 0.33 probability of receiving it during the time windows listed above. Both groups of participants, those in the Adaptive and Random arms were set to receive mental health tip messages, followed by the mood-prompt message, reminder messages (if needed), and feedback messages as described above.

Finally, participants in the Control arm of the study only received the mood-prompt message, reminder messages (if needed), and feedback messages. These participants did not receive the mental health support tip text messages.

### Outcome measures

The primary outcomes included measures of depression symptoms, 8-item Patient Health Questionnaire (PHQ-8), and anxiety symptoms, 7-item Generalized Anxiety Disorder (GAD-7) scale. These outcomes were collected through the baseline and exit Qualtrics surveys, administered upon study enrollment and at the end of the intervention period (day 61), respectively. In addition to PHQ-8/GAD-7, we asked about previous use of mental health services. Secondary outcomes included mood-prompt response rate, which we conceptualized as a proxy for engagement. Finally, we calculated participant-initiated discontinuation, or the proportion of participants replying ‘STOP’ to messages.

### Analysis

First, we examined participant demographics and mental health related variables by intervention arm and the overall sample. Next, we conducted a paired samples t-test to test for significant changes in the main outcomes, PHQ-8 and GAD-7 symptoms, pre-post intervention for the entire sample (hypothesis 1) as every participant in the study received mood-prompt and feedback messages. Next, we conducted an Analysis of Variance (ANOVA) and ANOVA-post hoc test to examine changes in main outcomes by intervention arm (Adaptive vs. Random vs. Control) (hypothesis 2). Finally, we conducted another ANOVA and post-hoc test to examine whether intervention mood-prompt response rate varied as a function of intervention arm (hypothesis 3). All analysis were performed in SPSS v. 28.

## Results

A total of 1717 individuals were screened to participate in this StayWell Trial between Dec 2021 and July 2022. Twenty six percent (*n* = 445) of participants were excluded from enrolling due to not passing phone verification procedures or incomplete enrollment information. Of those enrolled, 54 participants (3%) dropped out of the study by texting “Stop” (number of days in study: M = 16.6, SD = 14.7) and were excluded from subsequent analysis. Finally, due to an internal coding error in the adaptive arm of the study the data from *n* = 97 (5.6%) participants were removed from the trial as these users did not receive the reinforcement learning messages as intended.

The final sample included N = 1121 adults ages 18 to 79 (*M* = 38 years, *SD* = *10*.6). Participants were enrolled in the study an average of 58 days (SD = 3.3) and nearly three out of four (*n* = 814, 72.6%) were recruited via social media ads and the remaining via the HealthMatch portal (*n* = 307, 27.4%). The sample was diverse in terms of race/ethnicity, educational background, and employment status. The full demographics are reported in Table 2.

**Table 2:** Baseline Demographics

	Control n = 366	Random n = 377	Adaptive n = 378	Total N = 1121
**Age in years, Mean (SD)**	37.4 (10.8)	37.8 (10.5)	38.4 (10.6)	37.9 (10.6)
**Gender, n (%)**				
Female	281 (77%)	308 (82%)	283 (75%)	872 (78%)
Male	69 (19%)	53 (14%)	78 (20%)	200 (18%)
Transgender	11 (3%)	10 (3%)	11 (3%)	32 (3%)
Other / Not-listed	5 (1%)	6 (2%)	6 (2%)	17 (2%)
**Race/Ethnicity, n (%)**				
Asian or Pacific Islander	31 (9%)	38 (10%)	14 (4%)	83 (7%)
Black or African American	35 (10%)	34 (9%)	35 (9%)	104 (9%)
White or Caucasian	236 (65%)	254 (67%)	266 (70%)	756 (67%)
Latinx or Hispanic	24 (7%)	18 (5%)	27 (7%)	69 (6%)
Multi-Racial/Ethnic	33 (9%)	30 (8%)	30 (8%)	93 (8%)
Other / Refused to answer	7 (2%)	3 (1%)	6 (2%)	16 (2%)
**Education, n (%)**				
Some high school or less	8 (2%)	8 (2%)	8 (2%)	24 (2%)
High school graduate	40 (11%)	42 (11%)	58 (15%)	140 (12%)
Some college or technical school	123 (34%)	109 (29%)	118 (31%)	350 (31%)
College graduate	139 (38%)	149 (40%)	132 (35%)	420 (38%)
Graduate degree	56 (15%)	69 (18%)	62 (16%)	187 (17%)
**Employment, n (%)**				
Full time ( > 35 h/week)	140 (38%)	184 (49%)	162 (43%)	486 (43%)
Part-time ( < 35 h/week)	66 (18%)	54 (14%)	54 (14%)	173 (16%)
Homemaker	55 (15%)	42 (11%)	42 (11%)	139 (12%)
Unemployed	32 (9%)	29 (8%)	29 (8%)	90 (8%)
Disabled /on disability	36 (10%)	36 (10%)	55 (15%)	127 (11%)
Student	30 (8%)	19 (5%)	22 (6%)	71 (6%)
Retired / Other	7 (2%)	13 (3%)	14 (4%)	34 (3%)
**Difficulty paying basic needs**				
Not hard at all	86 (24%)	98 (26%)	85 (22%)	269 (24%)
Somewhat hard	184 (50%)	193 (51%)	174 (46%)	551 (49%)
Very hard	96 (26%)	86 (23%)	119 (32%)	301 (27%)
Mental health services history^*^				
Currently receiving	72 (37%)	53 (37%)	147 (39%)	272 (38%)
Received in the past	86 (44%)	62 (44%)	156 (41%)	304 (42%)
Never received	38 (19%)	27 (19%)	75 (20%)	140 (20%)

^*^n = 716.

Regarding adverse events, the system alerted us of 20 instances in which these words were used; however, 19 of these were false positives. False positives included instances in which the participant responded with words, such as “skills”, “my back hurts”, or even “San Diego”, as the system recognized the trigger words within the responses. For the other case the study PI sent a message to check in on the safety of the participant, which they confirmed, and research staff provided participant with suicide prevention and emergency/crisis line resources. On this particular case the participant continued in the study and exited as expected without additional triggers/flags. Specifically, the exchange was as follows:

Automated message: think about any time you were able improve your mood. Maybe you can try the same thing that helped you then.

Participant response: or maybe I could just die and put myself out of my misery,

PI response (manual): a response you sent was flagged as being possibly concerning. We wanted to check in to make sure you are OK and safe. Can you confirm?

Participant response: Yes

PI response (manual): we are glad to hear that you are OK. We want to remind you that these messages are not regularly monitored. If you need more support you can call the National Suicide Hotline 800-273-8255 or contact Crisis Text Line at 741741.

### Mental health outcomes

**Fig. 1:**
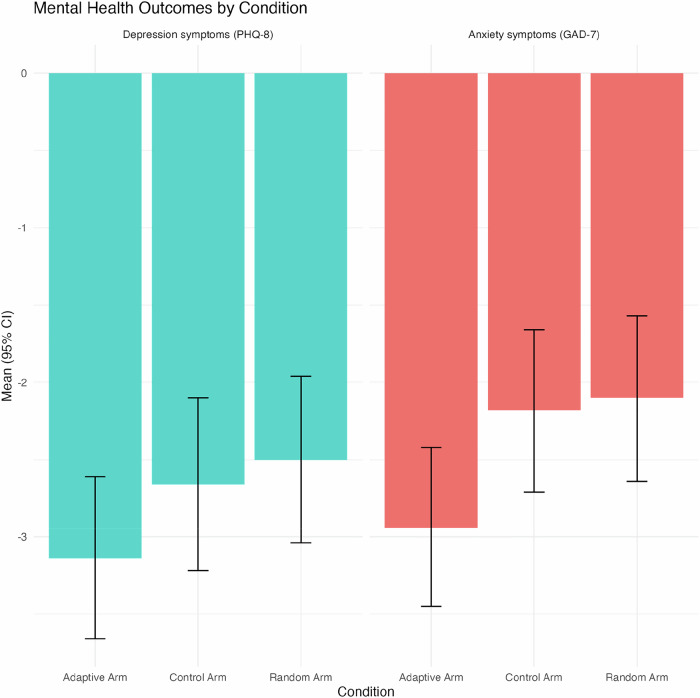
Mental Health Outcomes by Condition. This bar chart represents the average change in depression (PHQ-8: Patient Health Questionnaire – blue bars) and anxiety (GAD-7: General Anxiety Disorder – red bars) symptoms by intervention condition. The reductions represent improvements in symptoms and are calculated as the change from baseline (pre-intervention) to exit (post-intervention). The error bars represent the 95% confidence intervals.

On average, across all study arms, participants improved in their mental health outcomes supporting our hypothesis 1. Specifically, we observed a 25% reduction in depression symptoms (PHQ-8: Baseline *M(SD)* = 11.10 (5.93), Exit *M(SD)* = 8.34 (5.45), Change *M* (95% CI) = -2.76 (–3.07 to –2.45)); [t (948) = –17.39, *p* < *0.001*] and a 24% reduction in anxiety symptoms (GAD-7: Baseline *M(SD)* = 10.11 (5.67), Exit *M(SD)* = 7.70 (5.48), Change *M* (95% CI) = –2.40 (–2.71 to –2.10)); [t (948) = –15.53, *p* < *0.001*]. For hypothesis 2, when examining mental health outcomes by intervention arm the descriptive statistics showed the Adaptive arm had the greatest reduction in both PHQ-8 and GAD-7 symptoms relative to the Random and Control arms (Fig. 1); but the one-way ANOVA indicated the differences between arms were not statistically significant for PHQ-8 (F(2, 946) = [1.453], *p* = 0.234) or GAD-7 (F(2, 946) = [2.948], *p* = 0.053) (Table 3).

**Table 3:** Changes in mental health outcomes by intervention arm

	N	Baseline score, mean (SD)	Exit score, mean (SD)	Change from baseline, %	Mean difference (SD)	Mean Difference, 95% CI
**Depression symptoms (PHQ-8)**						
Adaptive	313	11.52 (6.07)	8.38 (5.67)	27.26	–3.14 (4.73)	–3.66 to –2.61
Random	322	10.89 (5.74)	8.39 (5.23)	22.96	–2.50 (4.93)	–3.04 to –1.96
Control	314	10.91 (5.98)	8.25 (5.48)	24.38	–2.66 (5.01)	–3.22 to –2.10
**Anxiety symptoms (GAD-7)**						
Adaptive	313	10.56 (5.71)	7.62 (5.57)	27.84	–2.94 (4.66)	–3.45 to –2.42
Random	322	9.81 (5.55)	7.71 (5.36)	21.41	–2.10 (4.87)	–2.64 to –1.57
Control	314	9.95 (5.73)	7.77 (5.55)	21.91	–2.18 (4.73)	–2.71 to –1.66

### Engagement Outcomes

On average, across all study arms, participants responded to 77% (Median = 0.90, SD = 0.29) of the mood-prompt messages sent to them. When comparing mood-prompt response rates by intervention arm, a one-way ANOVA test indicated there was a statistically significant difference between the intervention arms and mood-prompt response rates (F(2, 1118) = [5.185], *p* = 0.006). A Games-Howell Post Hoc test revealed the Control arm participants had statistically significantly higher mood-prompt response rates than both the Adaptive (Mean diff: –0.066, *p* = 0.006) and Random (Mean diff: –0.05, *p* = 0.043) arms (Table 4) therefore Hypothesis 3 (Adaptive arm having highest response rate) was not supported.

**Table 4:** Mood-prompt response rates by intervention arm

Intervention Arm	N	Mean (SD)	95% CI
Adaptive	378	74 (0.30)	71–0.77
Random	377	76 (0.29)	72–0.78
Control	366	81 (0.28)	78–0.83

### Post hoc exploratory analyses

We conducted a series of post hoc analysis to explore potential explanations for the unexpected findings from hypothesis 2 and 3. First, we examined if the intervention effects differed based on participants symptom severity at baseline by creating dichotomous variables, using a cut off score of 10 or higher on the PHQ-8 and GAD-7, and re-analyzing the intervention effects using one-way ANOVA for each new group. Next, we explored the impact of mood-feedback percentage on mood-prompt response rate. Finally, we analyzed descriptive statistics of intervention message categories to examine the reinforcement learning algorithm performance.

For participants with minimal to mild depressive symptoms (PHQ-8 < 10, *n* = 403) we found an 8% reduction in PHQ-8 scores [Baseline score: 5.59, Exit score: 5.11, mean change and 95% CI: -0.47 (-0.82, -0.13), t(402) = -2.72, *p* < 0.003] *and* found statistically significant differences on changes in PHQ-8 by intervention arm (F(2, 400) = [3.918], *p* = 0.021). A Games-Howell Post Hoc test indicated that participants in the Adaptive arm of the study had greater reductions in PHQ-8 scores relative to participants in the Random arm (Mean difference = -1.18, 95% CI: -2.26 to -0.10, *p* = 0.028). For participants in the moderate to moderately severe category (PHQ-8 ≥ 10, *n* = 546) we found a 29% reduction in PHQ-8 scores [Baseline score: 15.18, Exit score: 10.72, mean change and 95% CI: -4.45 (-4.88, -4.02), t(545) = -20.45, *p* < 0.001] but did not find statistically significant differences between intervention arms on changes in PHQ-8 (F(2, 543) = [0.409], *p* = 0.664).

For participants with minimal to mild anxiety symptoms (GAD-7 < 10, *n* = 478) we found a 11% reduction in GAD-7 scores [Baseline score: 5.33, Exit score: 4.72, mean change and 95% CI: -0.61 (-0.94, -0.28), t(477) = -3.66, *p* < 0.001] but the differences between intervention arms were not statistically significant (F(2, 475) = [2.114], *p* = .122). Similarly, for participants with moderate to severe anxiety symptoms (GAD-7 ≥ 10, *n* = 471) we found a 28% reduction in GAD-7 scores [Baseline score: 14.95, Exit score: 10.73, mean change and 95% CI: -4.22 (-4.68, -3.76), t(470) = -18.08, *p* < 0.001] but no statistically significant differences between intervention arms (F(2, 468) = [.569], *p* = 0.566).

We assessed the relationship between the percentage of time participants received mood-feedback messages on mood-prompt response rates among the full sample. A one-way ANOVA indicated that participants receiving mood-feedback messages 33% of the time had statistically significant greater mood-prompt response rate than participants receiving mood-feedback messages 100% of the time (0.80 vs. 0.76) (F(2, 1118) = [3.245], *p* = 0.039). There were no statistically significant differences between those receiving mood-feedback messages 66% of the time.

Finally, we analyzed the frequency of messages sent to the Random and Adaptive arms by each message category (Table 1) and observed a greater variability and distribution of message category selection among the Adaptive arm relative to the random arm of the study.

## Discussion

In this study, we evaluated whether the use of reinforcement learning algorithms, designed to personalize the timing and type of tip message sent to participants in the StayWell program (Adaptive arm), result in improved mental health outcomes (PHQ-8 and GAD-7) compared to not using a reinforcement learning algorithm (Random arm) and only a mood monitoring message (Control arm). On average, depression and anxiety symptomology scores decreased among participants in all arms (25% reduction in PHQ-8 and 24% reduction in GAD-7). We did not find support for the primary hypothesis that the adaptive arm would lead to improved outcomes overall. However, in post hoc analyses, for participants with mild depression scores (PHQ-8 < 10), the Adaptive arm of the study resulted in a greater reduction in PHQ-8 symptoms relative to the other two intervention arms; providing some evidence in support of the reinforcement learning algorithm to personalize the intervention for a subset of the sample. Finally, we found statistically significant differences in engagement rates (proportion of responses to mood-prompt messages) by intervention arm, with the participants in the Control arm responding to more messages than participants in the Adaptive and Random arms (contrary to our hypothesis). Finally, those receiving mood-prompt feedback 33% of the time were more engaged than those receiving feedback 100% of the time.

Our main finding that a reinforcement learning algorithm to adapt the delivery of the text messaging intervention did not improve mental health outcomes, is inconsistent with prior research which suggests RL can improve engagement, satisfaction, and outcomes^11,12,15^. The most surprising finding is that the control arm (only a mood monitoring message) yielded similar decreases in symptoms over time. Given these findings, it is important to consider possible explanations including 1) the mood monitoring control arm represents an active intervention and 2) the reinforcement learning algorithm as applied was not adequately personalized to yield significant differences.

It’s also possible that the mood monitoring component that is common to all of the interventions arms was strong enough to yield a clinical effect since symptoms went down for all groups including the “control” which only received mood monitoring messages. The common element of all intervention arms was sending users one daily mood self-monitoring text-message regardless of whether they received a tip message. In a recent secondary analysis of prior StayWell at Home intervention, we found that daily mood ratings increased during the first 2–3 weeks of the intervention and were maintained during the 60-day program regardless of message type or no message at all^16^; suggesting that ongoing daily mood-monitoring may be a powerful enough intervention to help participants improve their mental health outcomes. Tracking mood, triggers, thoughts, behaviors and emotional reactions is a common practice of CBT; thus, it may be that the ongoing reminder to check in with oneself, particularly when the SMS is delivered randomly throughout the day, helps participants raise awareness about their mental health and engage in self-regulation or other coping practices. Other research also finds that self-monitoring of symptoms is a key component driving a reduction in depression symptoms for various psychological interventions^17,18^. Thus, it is possible that simply mood monitoring messages are effective in decreasing depressive symptoms over time. Future work should assess mood-monitoring compared to no intervention or a wait list.

Another explanation for the lack of superiority of the adaptive condition could lie in our algorithm design decisions. There is evidence that reinforcement learning approaches can improve texting intervention efficacy in other health outcomes (e.g., physical activity, medication adherence)^11,12,15^. However, there are different ways that these algorithms can be designed, such as which model was used, which reward function is chosen, how missing data is handled, and a focus on algorithm performance versus effectiveness in the real world^19^. In particular, we opted for individually based models that would only use responses from individuals to personalize messages. While this could yield the most personalized intervention that uses only the individual’s data, it also requires more data and feedback from users to adequately personalize messages. This contrasts with group-based models that utilize data from all participants to determine which types of messages to send to participants. Though there were differences in the distribution of messages sent between the random and adaptive arms of the intervention, it is possible that the algorithm in this study did not guide sufficient personalization and may have benefitted from larger amounts of training data, a longer time frame for personalization to take place, or an adjustment from an individual-based model to a group-based model^19^. In further support of this explanation, there were greater decreases for the Adaptive arm, but they were not statistically significant, indicating that the effect was not strong enough. A scoping review also found that this type of work should conduct simulations prior to deployment and ideally assess how choices, such as a different model or reward function, may impact algorithm performance for text-based mental health studies^19^.

On the other hand, in our exploratory analysis of symptom levels as moderators, personalization via reinforcement learning in the adaptive arm of the study seemed to have an added benefit for participants with mild levels of depressive symptoms (PHQ-8 < 10). It’s possible that the information was more novel or that having milder symptoms allows these users to engage in the recommended practices. This difference merits further research and in particularly has implications for the prevention of diagnosed depression.

Simple text-messaging (SMS) based mental health interventions may be an effective low-cost, low-intensity, and rapidly disseminated type of intervention for improving population health, which is supported by other recent research^20^. Though the reinforcement learning algorithm did not significantly improve the intervention, overall, participants reduced their depression and anxiety scores following the 60-day texting intervention. We observed reductions in PHQ and GAD symptom severity for both participants with low symptom severity and moderate/high symptom severity at baseline, indicating that text-messaging might be useful both as health promotion, and for prevention of mental health disorders in at risk populations. Alhough we did not demonstrate that in this study, the strength of digital mental health interventions may be particularly situated in prevention of mental health problems, through increasing mental health skills and resilience. However, the implementation of preventive digital mental health in health systems globally is lacking, which is a priority for future research^21^.

As previously noted, it is possible the limitations of the algorithm may have impacted intervention efficacy. Additional research is needed to examine aspects of reinforcement learning algorithms that increase or decrease adaptiveness. For example, missing data for the mood-prompt message may have negatively impacted the performance of the reinforcement learning algorithm. We are not able to definitively assess the overall efficacy of the StayWell intervention compared to no intervention, as the Control arm provided users with a mood-monitoring text message which may be a strong intervention by itself. The intervention was designed to send daily mental health support text messages for participants; yet, as a research team we did not evaluate whether or not the research participants followed any of the recommendations provided to them. Finally, the over representation of female participants in the sample and the observed participant loss to follow up limit generalizability and may bias the findings.

It is important to develop and disseminate low cost and low intensity interventions for promoting mental health among the general and clinical populations. The results of this trial suggest that StayWell is a promising text-messaging intervention to achieve reductions in depression and anxiety among diverse populations. Additionally, it is possible that reinforcement learning algorithms can be improved and leveraged to further improve intervention effects; whether these would be treatment or prevention interventions. Finally, additional research is needed to examine the mechanisms of change in digital mental health interventions broadly, and text-messaging interventions specifically.

The various versions of the StayWell program, which delivers CBT/DBT informed text-messages and daily mood-monitoring text messages to users, resulted in reduced depression and anxiety symptoms among a general and clinical population. Receiving adaptive text messages via a reinforcement learning algorithm led to greater symptom decreases for those with mild depression symptoms at baseline. The use of text-message digital mental health interventions can help promote population health, help reduce symptom severity for patients waiting for care, and can help fill the gaps in existing mental health care and mental health promotion.

## Data Availability

The data sets generated and/or analyzed during this study are available from the corresponding author on reasonable request.
